# Cytokine-free directed differentiation of human pluripotent stem cells efficiently produces hemogenic endothelium with lymphoid potential

**DOI:** 10.1186/s13287-017-0519-0

**Published:** 2017-03-17

**Authors:** Yekaterina Galat, Svetlana Dambaeva, Irina Elcheva, Aaruni Khanolkar, Kenneth Beaman, Philip M. Iannaccone, Vasiliy Galat

**Affiliations:** 1 Developmental Biology Program, Stanley Manne Children’s Research Institute, Ann & Robert H. Lurie Children’s Hospital of Chicago, Chicago, IL USA; 2 Department of Microbiology and Immunology, Rosalind Franklin University of Medicine and Science, North Chicago, IL USA; 3 Department of Pathology, Stanley Manne Children’s Research Institute, Ann & Robert H. Lurie Children’s Hospital of Chicago, Northwestern University Feinberg School of Medicine, Chicago, IL USA; 4 Department of Pediatrics, Developmental Biology Program, Stanley Manne Children’s Research Institute, Ann & Robert H. Lurie Children’s Hospital of Chicago, Northwestern University Feinberg School of Medicine, Chicago, IL USA; 5 Department of Pathology, Developmental Biology Program, Stanley Manne Children’s Research Institute, Ann & Robert H. Lurie Children’s Hospital of Chicago, Northwestern University Feinberg School of Medicine, Chicago, IL USA; 6 Present Address: Department of Pediatrics, Division of Hematology & Oncology, Penn State Hershey College of Medicine, Hershey, PA USA

**Keywords:** Hemogenic endothelium, Pluripotent stem cells, Hematopoietic cells, Lymphocytes, Glycogen synthase kinase 3 inhibitor

## Abstract

**Background:**

The robust generation of human hematopoietic progenitor cells from induced or embryonic pluripotent stem cells would be beneficial for multiple areas of research, including mechanistic studies of hematopoiesis, the development of cellular therapies for autoimmune diseases, induced transplant tolerance, anticancer immunotherapies, disease modeling, and drug/toxicity screening. Over the past years, significant progress has been made in identifying effective protocols for hematopoietic differentiation from pluripotent stem cells and understanding stages of mesodermal, endothelial, and hematopoietic specification. Thus, it has been shown that variations in cytokine and inhibitory molecule treatments in the first few days of hematopoietic differentiation define primitive versus definitive potential of produced hematopoietic progenitor cells. The majority of current feeder-free, defined systems for hematopoietic induction from pluripotent stem cells include prolonged incubations with various cytokines that make the differentiation process complex and time consuming. We established that the application of Wnt agonist CHIR99021 efficiently promotes differentiation of human pluripotent stem cells in the absence of any hematopoietic cytokines to the stage of hemogenic endothelium capable of definitive hematopoiesis.

**Methods:**

The hemogenic endothelium differentiation was accomplished in an adherent, serum-free culture system by applying CHIR99021. Hemogenic endothelium progenitor cells were isolated on day 5 of differentiation and evaluated for their endothelial, myeloid, and lymphoid potential.

**Results:**

Monolayer induction based on GSK3 inhibition, described here, yielded a large number of CD31^+^CD34^+^ hemogenic endothelium cells. When isolated and propagated in adherent conditions, these progenitors gave rise to mature endothelium. When further cocultured with OP9 mouse stromal cells, these progenitors gave rise to various cells of myeloid lineages as well as natural killer lymphoid, T-lymphoid, and B-lymphoid cells.

**Conclusion:**

The results of this study substantiate a method that significantly reduces the complexity of current protocols for hematopoietic induction, offers a defined system to study the factors that affect the early stages of hematopoiesis, and provides a new route of lymphoid and myeloid cell derivation from human pluripotent stem cells, thus enhancing their use in translational medicine.

**Electronic supplementary material:**

The online version of this article (doi:10.1186/s13287-017-0519-0) contains supplementary material, which is available to authorized users.

## Background

Recently, substantial progress has been made in hematopoietic differentiation of human pluripotent stem cells (hPSCs) [1–4]. In combination with gene correction systems, hematopoietic differentiation provides an avenue for disease modeling [5, 6] and cellular therapies [7, 8]. Transplantation of hematopoietic stem cells (HSCs), which originate via the definitive hematopoietic program, offers potential treatment for a variety of hematological disorders [9, 10]. The hallmark of definitive hematopoiesis is the capacity of hematopoietic progenitors to produce cells of lymphoid lineage [11]. To date, the generation of various types of lymphoid cells, such as T lymphocytes ([12–14], reviewed [15]), natural killer (NK) cells [16], and induced NK (iNK) cells [17], and limited lymphoid B-cell potential [18] from hPSCs has been reported.

Nevertheless, the development of a fully defined system for generation of functional hematopoietic cell types, especially lymphocytes, remains a significant challenge. The identification of molecular mechanisms and factors driving the hematopoietic specification of various blood lineages from hPSCs is critical in overcoming this limitation [19]. Originally, hematopoietic induction was established by coculture with mouse stromal cells [20]. Currently, there are two main approaches to refining the conditions for induction of blood lineages. The first is based on selection of appropriate transcriptional regulators in gain-of-function experiments [21–23]. The second approach relies on the development of a cytokine regimen based on manipulations of the pathways of embryonic hematopoiesis [13, 24–26]. Several major stages were described during initial hematopoietic specification in the embryo [27] and in human pluripotent cells: APLNR^+^ primitive streak/mesendoderm precursors (mesenchymoangioblasts), VE-cadherin^+^/CD31^+^ angiohematopoietic progenitors, hemogenic endothelium (HE), and multipotential hematopoietic progenitors [1]. BMP4, Activin A, and bFGF are commonly used to induce mesodermal commitment of pluripotent cells. Subsequent stages of hemato-vascular differentiation are typically supplemented with combinations of VEGF, bFGF, IL-6, IL-3, IL-11, IPO, IGF1, and SCF [28].

The signaling landscape is crucial for fate determination at the initial stages of differentiation. Apparently, primitive and definitive hematopoietic specification segregates in the very early steps of hPSC differentiation and can be distinguished by Glycophorin A (CD235a) expression by the second day after induction. In particular, it was shown that specification of definitive progenitors (CD235a^−^) requires Wnt–b-catenin signaling [11]. The glycogen synthase kinase 3 (GSK3) inhibitor CHIR99021 is a known Wnt agonist [29]. GSK inhibition was shown to induce hPSC differentiation to vascular progenitors [30, 31] and definitive hematopoietic cells in cell aggregates [11]. In combination with an OP9 coculture system, this was also shown to facilitate the development of hematopoietic progenitors in primates [32].

Here, we demonstrate that exposure of hPSCs growing in adherent culture to the inhibitor of glycogen synthase kinase 3 (GSK3 inhibitor) induces their specification to a definitive HE without the addition of any cytokines. Once transferred to the appropriate culture conditions, these endothelial progenitors are capable of producing lymphoid cells (NK, T and B cells) as well as various cells of myeloid lineage. This finding provides a significant advance in defining critical components for the induction of definitive hematopoiesis in vitro. By reducing the complexity of hematopoietic induction, this offers a defined system to study the factors affecting hematopoiesis.

## Methods

### iPSC derivation

The iPSC-SR2 line was derived from MRC-5 fibroblasts (ATCC) by overexpressing Oct4, Sox2, Nanog, and cMyc using retroviral vectors (pMXs-cMyc, pMXs-Klf4, pMXs-hOct3-4, and pMXs-Sox2; Addgene) [33]. The retroviral vectors were produced by transient transfection of 293 T cells. The fibroblasts were incubated in the viral supernatants containing 5 μg/ml polybrene (Sigma) for 4 h. The transduced cells were then incubated for 3 weeks until development of the pluripotent clones. After isolation the clones were grown in mTeSR1 medium (Stem Cell Technologies) on a Matrigel® substrate (BD Biosciences). The cultures were split mechanically using the StemPro EZ Passage tool (Invitrogen).

### OP9 coculture induction

hPSCs were maintained on irradiated embryonic mouse fibroblasts (MEFs) in DMEM–F12 medium supplemented with serum knockout replacement, L-glutamine, nonessential amino acid, and basic FGF (4 ng/ml). On day 7 after the last passage, hPSCs were treated with collagenase IV (1 mg/ml) for 5 min. Clumps of hPSCs were collected and transferred into OP9 stromal cells supplemented with 10 ml of MEM-Alpha medium containing G-Max (Invitrogen) and 20% FBS (HyClone). Next day, medium was replaced with 20 ml of differentiation media containing 10% FBS (HyClone), MTG (100 μM), and ascorbic acid (50 μM) and thereafter was changed every 4 days. The cells were collected on day 8 of differentiation.

### CHIR99021 induction

HE differentiation was established by a monolayer induction protocol. Basically, as described previously [30], single cells were plated on 60-mm culture dishes coated with Matrigel® and cultured overnight in iPS-Brew or mTeSR1. Differentiation was induced with an induction media containing advanced DMEM/12 (Life Technologies), glutamax (2.5 mM), ascorbic acid (60 μg/ml), and CHIR990921 (6 mM) added on day 0. On day 2 of induction CHIR990921 was removed from the media. The cells were collected on day 5 of differentiation. HE cells were isolated with positive selection of CD34^+^ cells on magnetic columns (Miltenyi Biotec).

### Endothelial cells

Endothelial cells (EC) were cultured in Vasculife media (Life Line Cell Technologies). The endothelial potential of EC was evaluated using a tube formation assay, immunostaining for von Willebrand Factor (vWF), and acetylated-low density lipoprotein (Ac-LDL) uptake. Regarding the tube formation assay, EC were seeded onto a 12-well plate coated with Matrigel® (BD Biosciences) at a density of 4.5 × 10^4^ per well and incubated in Vasculife medium overnight at 37 °C, 5% CO_2_. Ac-LDL uptake was performed using a commercial kit (Biomedical Technologies) in accordance with the manufacturer’s instructions. EC were seeded onto 0.1% gelatin-coated (Sigma) plates at a density of 2.5 × 10^5^ cells per well in a six-well plate. Ac-LDL was diluted to 10 μg/ml in complete Vasculife medium and added to the cells for 3 h. The cells were then washed with Vasculife medium and analyzed via florescent microscopy. Expression of vWF was confirmed by staining EC using primary anti-hvWF A2 antibody (R&D systems) and the appropriate secondary antibody (Alexa-Fluor).

### Hematopoietic colony forming assay

The hematopoietic colony forming assay was performed in MethoCult H4435 medium (Stem Cell Tech) supplemented with Flt-3 L, IL-7, IL-3, SCF, and TPO (PeproTech, NJ, USA).

### Cytospin analysis

The cells were collected into 150 μl of PBS containing 10% FBS, placed into a Shandon EZ Single Cytofunnel (Thermo Scientific, MA, USA), and spun at 1000 rpm for 5 min. The slides were then air dried, fixed with methyl alcohol, and stained with Wright Stain (Fisher Scientific, MA, USA).

### Lymphocytes

Lymphocytes were derived by coculture of progenitor cells with OP9-DLL4 stromal cells [11]. On day 5 of monolayer culture, cells were treated with 0.05% Trypsin–EDTA (Gibco) and plated on semi-confluent dishes of OP9-DLL4, supplemented with differentiation medium containing IL-7 (10 ng/ml), IL-3 (5 ng/ml), hFlt-3 L (50 ng/ml), and hSCF (10 ng/ml) (all cytokines purchased from PeproTech). On day 3 of OP9-DLL4 coculture, semi-adherent cells were collected and passaged into a new dish with OP9-DLL4 cells. After this, floating cells were passaged to new plates every 4–5 days for a period of 14–30 days.

### Microscopy

Confocal images were acquired using an LSM 510 META Laser Scanning Microscope system (Zeiss, Thornmood, NY, USA). By varying the width of the pinhole of the detectors, the observed fluorescence was localized to a known thickness of observed tissue and the depth of field of the transmitted and DIC images was adjusted. Scale bars were integrated into the image during acquisition. Epifluorescence images were acquired on a Leica DM IRB inverted microscope system (Wetzlar, Germany) using a Hamamatsu ORCA-ER digital camera (Hamamatsu City, Japan) controlled with Improvision Openlab software version 5.0.2 (Lexington, MA, USA). Scale bars were calibrated to each objective magnification and added after acquisition. Light microscopic images were acquired with a Nikon D100 (Tokyo, Japan) digital SLR camera on an inverted Leica DM IRB microscope.

### Immunocytochemistry

The cells were washed with PBS, fixed with 4% paraformaldehyde (PFA) for 5 min, and permeabilized with 0.1% Triton X-100 for 5 min. After 30-min incubation at 37 °C with blocking solution (Protein Block; Dako), cells were incubated with primary antibodies (1:100) for 1 h at room temperature. Secondary antibody (1:250) was incubated for 30 min at 37 °C. Mounting medium containing DAPI (Life Technologies) was used for counterstaining nuclei. An alkaline phosphatase substrate kit (Vector Laboratories Inc.) was used for detection of alkaline phosphatase activity according to the manufacturer’s instructions.

### Flow cytometry

Antibodies CD31, CD34, and CD144 were purchased from Miltenyi Biotec. The cells were stained with the appropriate conjugated antibodies for 30 min at 4 °C. Cells were washed in a 0.5% BSA/PBS solution and analyzed via FACSCalibur (BD Biosciences, San Jose, CA, USA). Antibodies directed against CD3, TCRαβ, and TCRγδ as well as the murine isotype-control antibody IgG1,k used to assess background fluorescence for TCR expression were obtained from BD Biosciences (San Jose). The samples that assessed TCR expression were acquired on a FACS-Canto II flow cytometer (BD Biosciences, San Jose) and analyzed using FlowJo software version 10.1 (Tree Star Inc., Ashland, OR, USA)

For flow cytometry analysis of NK cell differentiation, the cells were labeled with a combination of monoclonal antibodies (mAbs) including CD45 Krome Orange (Beckman Coulter) and CD56 PE, CD16 PE-Dazzle, CD15 PerCP-Cy5.5, and CD94 APC (all from Biolegend, San Diego, CA, USA). For the isotype controls, specific mAbs were substituted for corresponding nonspecific IgG isotypes. LIVE/DEAD Fixable Violet stain (ViVID; Invitrogen, Eugene, OR, USA) was included to discriminate dead cells. Labeling was performed in 96-well U-bottom plates using 2.5 × 10^5^–3 × 10^5^ cells in 50 μl of PBS per well. After 25-min incubation with mAbs at 4 °C, cells were washed twice, subsequently transferred into 5 ml Falcon tubes (BD Biosciences, Bedford, MA, USA), and processed on LSR-II (BD Biosciences, San Jose). Compensation controls were prepared using an AbC anti-mouse bead kit for mouse mAb capture and ArC amine reactive compensation bead kit for ViVID (both kits from Invitrogen), according to the manufacturer’s instructions. Data analysis was performed using FlowJo software (Tree Star Inc.).

## Results

### GSK3 inhibition gives rise to hemogenic endothelium in the absence of cytokines or mouse stromal feeder cells

**Fig. 1:**
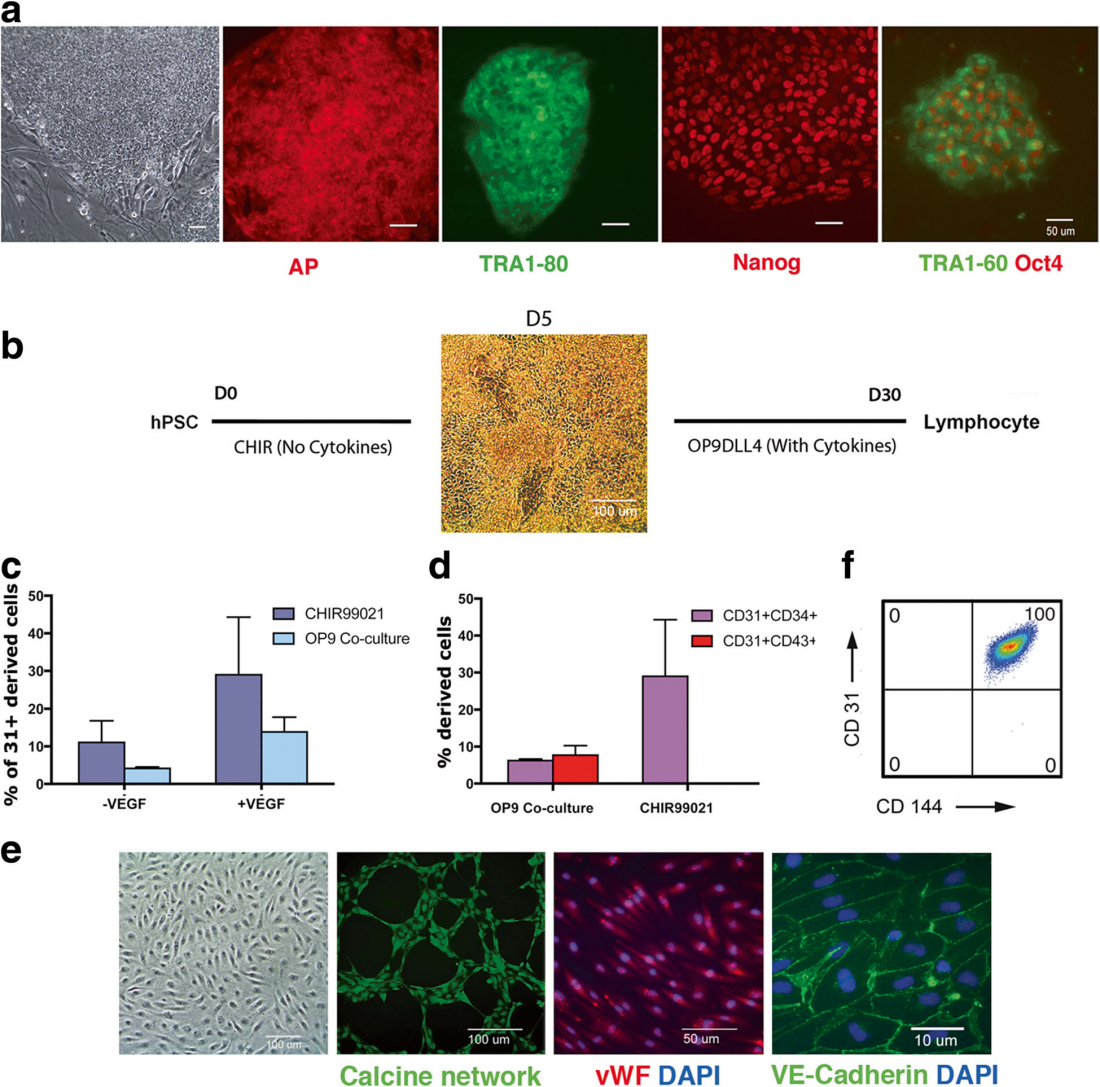
Derivation of CHIR99021-induced HE and iPSC-SR2 characterization. **a** Phenotypic characterization of iPSC-SR2: phase-contrast image of the cells when grown on MEFs. Expression of alkaline phosphatase (*AP*, *red*) and of characteristic pluripotency genes (TRA 1-80 (*green*), Nanog (*red*), and TRA 1-60 (*green*) and OCT4 (*red*) overlay) assessed by immunostaining. **b** Timeline of lympho-myloid differentiation and representative phase-contrast image of hPSCs differentiating to vascular progenitors on day 5 after induction with the small molecule CHIR99021. **c** Induction efficiency of CHIR99021-induced vs OP9 cocultured pluripotent cells as a percentage of CD31^+^ cells. *Error bars* are SEM of three independent experiments. **d** Homogeneity of CD31^+^ double-positive cells obtained from CHIR99021 induction vs heterogeneous population obtained from OP9 coculture. **e** At day 5, CD31^+^ cells were enriched with MACS selection column and quantified by flow cytometry for CD31 and CD144 expression. Phenotypic and functional characterization of isolated cells: phase-contrast image of cells when grown in endothelial medium, tube formation assay with calcein AM staining, expression of vWF assessed by immunostaining (*red*) overlay with Dapi, and expression of VE-cadherin assessed by immunostaining. **f** Cytometric flow analysis demonstrating homogeneity of CD31^+^ and CD144 coexpression by vascular cells at passage 3. *D* day, *hPSC* human pluripotent stem cell, *vWF* von Willebrand factor

Hemogenic endothelium (HE) can be produced by coculture with OP9 stromal cells or by the EB induction method (reviewed by Slukvin [19]). In this study we use embryonic stem cell lines H1 and H9 as well as induced pluripotent stem cell (iPSC) line SR2 to show that a large quantity of HE, capable of myeloid and lymphoid differentiation, can be produced by GSK3 inhibition in the absence of hematopoietic cytokines or mouse stroma. The utilized hPSC line iPSC-SR2 was derived from MRC-5 fibroblasts and shown to express a set of characteristic pluripotency markers (Fig. 1a), had a normal karyotype (Additional file 1: Figure S1A), and met the criteria established for pluripotent cells based on the evaluation of its whole genome expression pattern using a PluriTest platform (Additional file 1: Figure S1B). Additionally, hPSC line iPSC-SR2 was shown to differentiate into derivatives of three main embryonic lineages in vitro: endoderm [34], ectoderm [35], and mesoderm [31, 36].

The timeline for the experiment is shown in Fig. 1b. HE was derived on day 5 of differentiation and then cocultured with OP9-DLL4 and various cytokines in order to assess its hematopoietic potential. Particularly, the differentiation of hPSCs cultured in mTESR1 or iPSC-Brew was induced by culture with glycogen synthase kinase 3B (GSK3) inhibitor CHIR99021 (6 mM) for 2 days. The inhibitor was then removed and the cells were subsequently cultured in Advanced DMEM/F12, supplemented with ascorbic acid for 3 more days. HE development was assessed by FACS analysis as the percentage of CD31^+^ cells, on day 5 of differentiation. The results were compared to the OP9 coculture method. As shown in Fig. 1b, although with some variation, the cells cultured via the monolayer protocol generated more CD31^+^ cells than those cultured on OP9 in the presence or absence of VEGF, which is known to enhance hematopoietic cell differentiation (Fig. 1c). Notably, whereas the cells generated on OP9 included CD31^+^CD43^+^ and CD31^+^CD34^+^ suggesting that hematopoietic and endothelial progenitors are produced, the monolayer induction protocol CD31^+^ cells were all double positive for the marker CD34^+^ (Fig. 1d) and generated no CD43^+^ cells (data not shown). The absence of CD43^+^ cells was also noted by Sturgeon et al. [11], who studied hematopoiesis induced with cytokines in cell aggregates and did not find CD43^+^ cells in the presence of CHIR99021. They proposed that CHIR99021 inhibits primitive hematopoiesis and promotes definitive hematopoiesis, which expresses CD43^+^ at later stages of development. Overall, using our CHIR99021 induction method, we were able to generate >4 × 10^5^ CD31^+^CD34^+^ HE cells per 1 × 10^5^ hPSCs plated. When isolated and propagated in adherent conditions, these CD31^+^CD34^+^ progenitors gave rise to mature endothelium similar to results described by Lian et al. [30]. The endothelial characteristics of ECs were confirmed with CD31^+^/VE-cadherin coexpression (Fig. 1f) and demonstrated in functional assays such as Ac-LDL uptake (not shown), the tube formation assay and immunostaining for vWF (1e).

These data thus demonstrate that the method introduced here, which eliminated the need for cytokines, culture on MEFs, and expensive media, does robustly produce CD31^+^CD34^+^ double-positive HE.

### Myeloid potential assessment

**Fig. 2:**
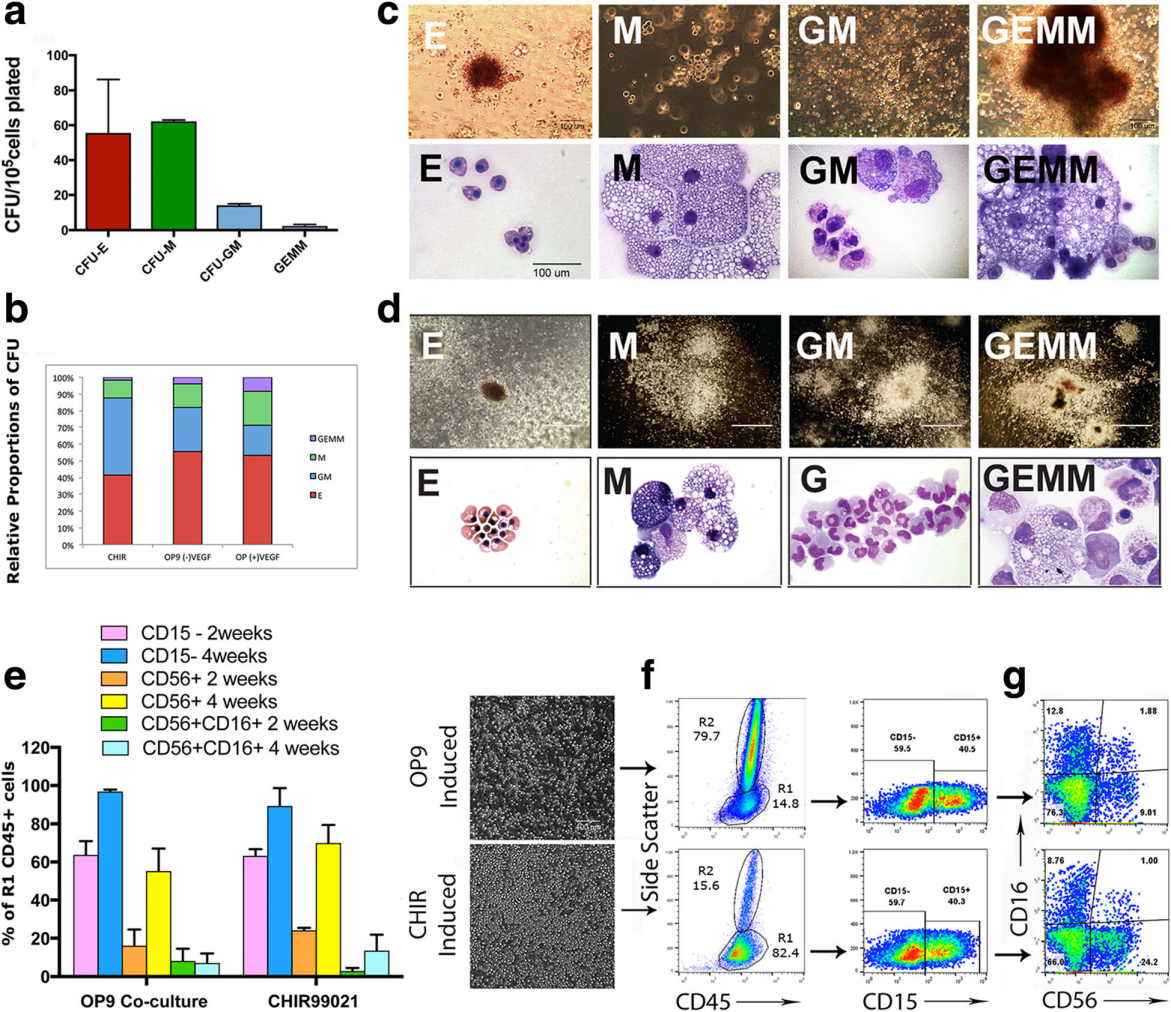
Myeloid and NK-lymphoid potential assessment of CHIR99021-induced HE and OP9 coculture-derived progenitors. **a** Myeloid potential assessment of CHIR99021 derived HE by quantitative colony forming unit (*CFU*) assay. *Error bars* are SEM. **b** Relative proportion of the different types of colonies formed after CHIR99021 induction compared to OP9 coculture induction with and without VEGF. **c** Phase-contrast images of colony morphology and corresponding cytospin images of E, M, GM, and GEMM colonies formed in semi-solid medium after CHIR99021 induction. **d** Phase-contrast images of colony morphology and corresponding cytospin images of E, M, GM, and GEMM colonies formed in semi-solid medium after OP9 co-culture induction. **e** Comparison of CHIR99021-induced and OP9 coculture-induced flow percentages of CD15^–^, CD56^+^, and CD56^+^CD16^+^ cells after 2 and 4 weeks of culture in lymphoid differentiation medium. The analyzed cells are gated on CD45^+^ fraction with side scatter characteristics for lymphoid-like cells. *Error bars* are SEM. **f** Representative phase-contrast images of cells floating on OP9-DLL4 and representative flow analysis showing CD45^+^ cells. *R1*, region of CD45^+^ cells with side scatter characteristics of lymphoid-like cells. **g** Representative flow cytometry analysis showing the percentage of CD15^–^ lymphoid fraction of differentiating cells and representative flow analysis showing the expression of CD56^+^ and CD16^+^ cells gated on CD15^–^ fraction. *E* erythroid, *M* macrophage, *G* granulocyte component of the GM colony, *GM* granulocyte/macrophage, *GEMM* granulocyte, erythroid, monocyte and megakaryocyte colonies

To demonstrate the myeloid potential of CD31^+^CD34^+^ HE derived by this monolayer culture method, we assayed the hematopoietic colony forming potential. The cells were briefly cocultured with OP9 and then plated in methylcellulose media containing Flt3, IL-7, IL-3, SCF, and TPO. The OP9 coculture was a necessary step due to the fact that the hematopoietic commitment of CD31^+^CD34^+^ progenitors is not revealed unless the cells are cultured in hemogenic endothelial conditions or with Notch ligand-expressing stromal cells [11]. We found that the cells formed erythroid (CFU-E), granulocyte/macrophage (CFU-GM), macrophage (CFU-M), and multilineage progenitor (CFU-GEMM) colonies (Fig. 2a). Notably, the quantitative analysis of the colony forming units revealed a trend for a relative proportion of colonies formed after induction skewed toward the CFU-GM compared with the relative proportion of colonies formed after OP9 coculture, which contained the largest number of CFU-E colonies (Fig. 2b). Colonies that formed after CHIR99021 induction and OP9 coculture induction were identified using microscopic images of colony morphologies and cytospin analysis of collected colonies (Fig. 2c, d).

### CHIR99021-induced hemogenic endothelium is capable of efficient natural killer differentiation

The capacity to produce lymphocytes is a hallmark of definitive hematopoiesis [11]. Lymphoid cell generation from hPSCs in vitro is a long two-step process (40–60 days). Hematopoietic induction is usually performed using stromal cells (e.g., OP9 or M210-B4) or using embryoid bodies and cytokines. The second step for T cells includes using OP9 cells expressing the NOTCH ligands d-like 1 (DLL1) or d-like 4 (DLL4) [12–14], reviewed by Smith et al. [15]. For NK cells, EL08-1D2 stromal cell coculture and/or cytokines are used [16, 37, 38]. No efficient conditions have so far been reported for B-cell differentiation from hPSCs.

To evaluate the lymphocyte-generating capabilities of HE cells produced on day 5 after CHIR99021 induction, we plated the cells onto OP9-DLL4 in MEM-Alpha medium containing growth factor cocktail for lymphoid cell differentiation. The medium contained MTG, ascorbic acid, and IL-3, IL-7, SCF, and FLT3. The results were assessed by FACS analysis at approximately 2 weeks (when the cells exhibited the most rapid proliferation activity) and 4 weeks of OP9-DLL4 coculture. The results of flow experiments are summarized in Fig. 2e. The scatter of analyzed floating CD45^+^ cells showed two distinct cell populations (R1 and R2), where R1 scatter was characteristic for lymphoid cells. The gating strategy (Additional file 1: Figure S1C) revealed that all lymphoid cells were contained within the R1 population. Overall, flow cytometric analysis demonstrated that, with little variation, CHIR99021-induced HE generated a much greater R1 CD45^+^ fraction (~80%) with side scatter characteristic for lymphoid cells which contained an NK cell population compared to that induced by OP9 coculture (~14%) (Fig. 2f).

In order to distinguish between lymphoid cells and the cells of myeloid lineage we employed CD15, a myeloid marker that is expressed by monocytes and granulocytes but not lymphoid cells [39]. We have found that about 60% of the cells within the CD45^+^ fraction with side scatter characteristic for lymphoid cells were negative for CD15 at 2 weeks of differentiation. After 4 weeks of OP-DL4 coculture, >97% of all cells were negative for CD15.

The analysis of cells gated on the CD15^–^ lymphoid fraction showed that after 2 weeks of differentiation CHIR990921-induced HE developed a much larger population of CD56^+^ NK cells (~25%) compared to OP9-induced cells (~15%), and the fraction of CD56^+^CD16^+^ double-positive NK cells, the major population subset of NK cells at this stage of development, was small for both CHIR990921 and OP9-induced cells (Fig. 2g). Notably, CHIR990921-induced progenitors yielded a larger CD56^+^CD16^–^ fraction, the minor population of NK cells that is present in peripheral blood. By 4 weeks of differentiation, NK cells derived using the OP9 coculture method remained mostly CD56^+^ single positive, while ~20% of lymphoid cells derived from HE induced via CHIR99021 became double positive for CD56^+^CD16^+^. This result suggests that CHIR99021-induced progenitors may mature more quickly in culture.

These findings demonstrate that the HE derived by the method described here is capable of NK-lymphoid cell differentiation in conditions that are suitable for various types of immature NK cell derivation and potentially can be manipulated for large-scale mature NK cell production for clinical use as well as disease modeling and drug screening.

### CHIR99021-induced hemogenic endothelium is capable of T-cell and B-cell differentiation

**Fig. 3:**
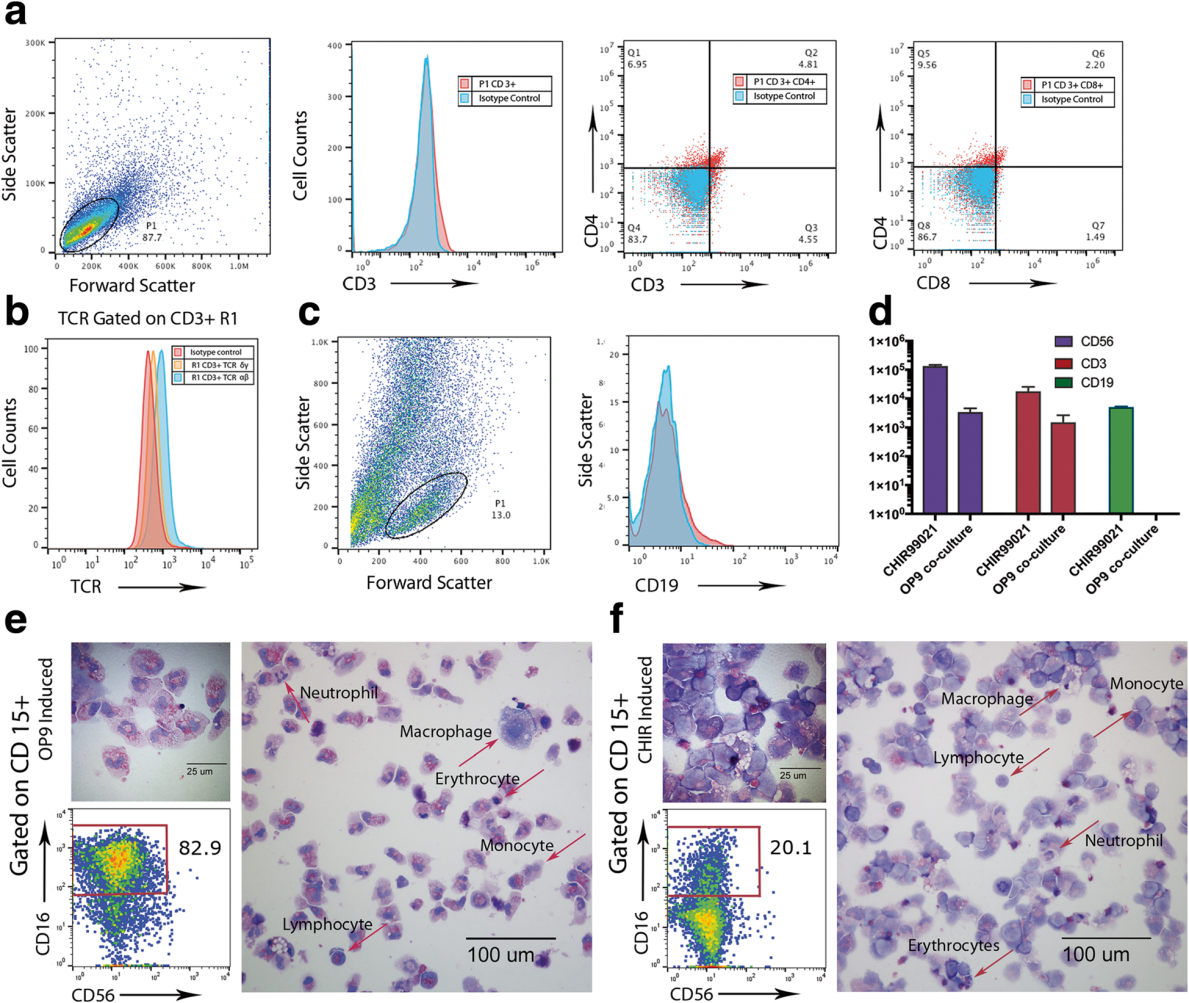
T-lymphoid, B-lymphoid, and myeloid potential assessment of CHIR99021-induced HE and OP9 coculture-derived progenitors. **a** Representative flow cytometry analysis showing the differentiating cells gated on the lymphoid fraction containing the CD3^+^ T-cell population and the percentage of CD4^+^CD8^+^ double-positive T cells after 3 weeks of differentiation. **b** Representative flow cytometry analysis showing TCRαβ and TCRγδ rearrangements gated on CD3^+^ cells after 4 weeks of differentiation. **c** Representative flow cytometry analysis demonstrating the percentage of cells expressing B-lymphoid potential (CD19). **d** Yield of CD56^+^, CD3^+^, and CD19^+^ cells per 1 × 10^5^ cells plated. *Error bars* are SEM. **e** Representative cytometric analysis of cells derived after OP9 coculture induction gated on CD15^+^ myeloid fraction, and cytospin images of floating cells showing a variety of hematopoietic cell types. **f** Representative cytometric analysis of cells derived after CHIR99021 induction gated on CD15^+^ myeloid fraction, and cytospin images of floating cells showing a variety of hematopoietic cell types. *TCR* T-cell receptor

In order to assess the T-cell and B-cell potential of CHIR99021-induced HE, we used flow cytometry to analyze the CD45^+^ cells gated on the lymphoid fraction for T-cell and B-cell markers. The analysis demonstrated that after 3 weeks of differentiation on OP9-DLL4, up to 10% of these cells began to express CD3. This result was comparable with the percentage of CD3^+^ cells we obtained via the OP9 coculture induction method (data not shown). Up to 3% of CD3^+^ cells were also double positive for markers CD4 and CD8 (Fig. 3a). Furthermore, most lymphoid CD3^+^ T-cell progenitors underwent some degree of T-cell receptor (TCR) rearrangement generating predominantly αβ (61%) and some γδ (29%) expression after 4 weeks of differentiation (Fig. 3b), indicating that our OP9-DLL4 coculture system produces T cells that undergo initial stages of maturation. In order for T-cell progenitors to progress to mature phenotypes, a specific antigen stimulation system should be implemented [40].

Notably, CHIR99021 induction promotes differentiation of B cells (Fig. 3c). Currently, there are very few studies, conducted using MS-5 stroma, that demonstrate a limited lymphoid B-cell potential of pluripotent cells in their differentiation systems [18].

Overall, using our CHIR99021 induction method we were able to obtain ~2.5 × 10^5^ CD56^+^ NK-lymphoid cells, ~3.5 × 10^4^ CD3^+^ T-lymphoid cells, and ~1.0 × 10^4^ B-lymphoid cells per 10^5^ cells plated, as compared to the OP9 coculture induction method, which generated only ~6.7 × 10^4^ CD56^+^ NK-lymphoid cells, ~3.0 × 10^3^ of CD3^+^ T-lymphoid cells, and no B-lymphoid cells (Fig. 3d).

### Myeloid population produced by CHIR990921-induced hemogenic endothelium is different from myeloid population produced with OP9 coculture

To identify the myeloid cells remaining in CD15^+^ myeloid populations, we analyzed hematopoietic cultures growing in suspension by flow cytometry and morphological analysis of Wright-stained cytospins. We found that these cells did not express CD56 or CD94 (data not shown), but had variable expression of CD16, indicating that the population comprised a variety of monocytes and granulocytes. Interestingly, at 2 weeks the OP9 coculture induction protocol yielded a population of cells that was predominantly CD15^+^CD16^+^ (Fig. 3e), while the population of CHIR99021-induced cells was much less homogeneous (Fig. 3f), suggesting that CHIR99021 induction enhances the development of multipotential progenitors resulting in a wider spectrum of myeloid cells.

## Discussion

Our study offers an important insight into the early stages of hematopoietic specification of pluripotent stem cells. The data show that multipotential hematopoietic progenitors can be established in a relatively simple adherent system by promoting the stages of primitive mesendoderm precursors, angiohematopoietic progenitors, and HE with a GSK3 inhibitor, which points to the key role of Wnt–b-catenin signaling in definitive hematopoietic development. This finding provides an efficient method of hPSC differentiation to HE that is capable of definitive hematopoiesis, without the use of cytokines in a defined serum-free system. Further studies are needed to clarify whether GSK3 inhibitor reflects the requirement of the hematopoietic niche in vivo or simply creates a benefit promoting hematopoiesis in tissue culture.

There are several reports of hematopoietic differentiation of hPSCs with the aid of the murine-derived OP9 stromal cell coculture, or feeder-free EB formation systems [20, 41–43]. The overall efficiency of hematopoietic development using the stromal cells is relatively limited [16], whereas the EB induction method requires the use of expensive cytokines and complicates determination of hematopoietic–stromal cell interactions. Furthermore, both of these methods have undefined factors produced either by the stromal cell or the 3D interaction. Using the OP9 coculture protocol makes it impossible to control the cytokines secreted by the stromal cells, and the sphere-like structure of the embryoid bodies complicates determination of hematopoietic niche components. The monolayer CHIR99021 induction method described here allows control of all of the variables aside from the cell line response. Differentiation in defined serum-free monolayer culture was reported previously, but until now the “monolayer” system only allowed hPSCs to differentiate into “primitive” hematopoietic progenitor cells (HPCs) [44] and development of T cells was not attained [43].

Monolayer induction based on GSK3 inhibition, described here, yields a large number of HE cells that can then differentiate further into various types of myeloid and lymphoid lineages. Myeloid potential was demonstrated by a range of myeloid CFUs, consisting of CFU-E, CFU-GM, CFU-M, and CFU-GEMM, as well as CD15^+^ cells produced after 2 weeks of OP9-DLL4 coculture. Substantial numbers of NK cells and some T cells and B-cell precursors that developed after subsequent coculture of HE with OP9-DLL4 demonstrated the strong lymphoid potential of this differentiation system. Notably, this system favors NK cells, which perhaps reflects a bias of the formulation of the cytokine cocktail at the OP9-DLL4 differentiation stage. A similar observation that lymphoid development of hPSC-derived cells strongly favors the NK-cell lineage was reported previously by the Kaufman laboratory [45].

Although it is evident that the cytokines may be omitted at the initial stages of progenitor development, they may potentially change the lineage preference or functionality of mature phenotypes. For instance, Pearson et al. [46] demonstrated engraftment of Flk1^+^ mesodermal cells generated from mouse ESCs. The authors believe that the lack of SCF, IL-3, and IL-6 hematopoietic cytokines in the culture prevented differentiation and allowed for the accumulation of engraftable cells.

Recently several promising applications have been shown for hPSC-derived lymphocytes, such as adoptive cell transfer immunotherapy (ACT) for treatment of patients with cancer [12, 47], viral diseases [48], and regaining self-tolerance by delivering regulatory T cells [49]. In turn, hPSC-derived NK cells exhibit potent antitumor activity without the need for human leukocyte antigen matching and without prior antigen exposure [16]. These cells were also recently employed for treatment of ovarian cancer in a mouse model [50]. Our study substantiates an efficient, controllable, and less expensive approach to hematopoietic cell derivation compared to existing technologies, which may fulfill a growing need for translational medicine for a scalable production of lymphoid and myeloid cells.

## Conclusion

This work demonstrates that specification of definitive hematopoietic progenitors from hPSCs can proceed to the HE stage in the presence of a small molecule GSK3 inhibitor without any additional cytokines. The defined system presented here permits an investigation of the influences of cytokines during the early stages of hematopoietic cell fate commitment and development. By combining an enhanced monolayer induction method, which generates large quantities of HE, with a mouse stromal coculture protocol, we have achieved an efficient differentiation of hPSCs along various lympho-myeloid lineages.

## Additional file

Additional file 1: Figure S1. showing characterization of iPSC-SR2 and gating strategy for lymphoid cell analysis. (**A**) Normal karyotype of iPSC-SR2. (**B**) Pluripotency assessment results of iPSC-SR2 by PluriTest. (**C**) Gating strategy for identifying various lymphoid cells in differentiating cultures after CHIR99021 induction and OP9 coculture induction. R1 representing CD45^+^ showing CD15, CD56, CD16, or CD94 expression, while none of these markers are present on cells in R2. (TIF 10931 kb)

## Abbreviations

ACT: Adoptive cell transfer immunotherapy
CFU: Colony forming units
E: Erythroid
EB: Embryoid body
EC: Endothelial cells
GEMM: Granulocyte, erythrocyte, monocyte, megakaryocyte
GM: Granulocyte/macrophage
GSK3: Glycogen synthase kinase 3
HE: Hemogenic endothelium
HPC: Hematopoietic progenitor cell
hPSC: Human pluripotent stem cell
HSC: Hematopoietic stem cell
iNK: Induced natural killer
iPSC: Induced pluripotent stem cell
M: Macrophage
NK: Natural killer
PFA: Paraformaldehyde
TCR: T-cell receptor
