# Supplementary figures and images for: Cytokine-free directed differentiation of human pluripotent stem cells efficiently produces hemogenic endothelium with lymphoid potential
Source: Stem Cell Res Ther. 2017 Mar 17;8:67. doi: 10.1186/s13287-017-0519-0 (PMC5356295; doi:10.1186/s13287-017-0519-0)

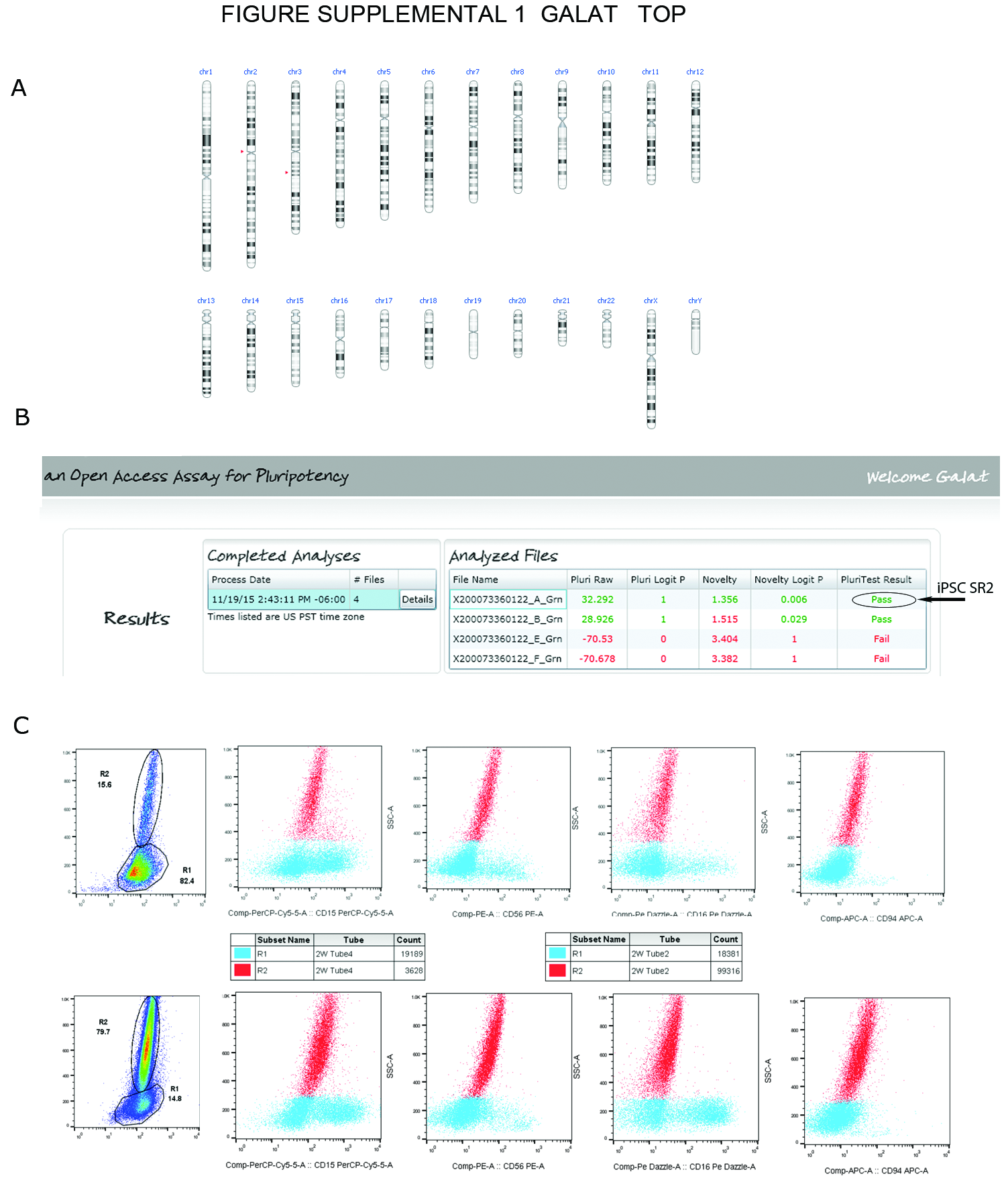

Supplement: Additional file 1: Figure S1. — showing characterization of iPSC-SR2 and gating strategy for lymphoid cell analysis. (A) Normal karyotype of iPSC-SR2. (B) Pluripotency assessment results of iPSC-SR2 by PluriTest. (C) Gating strategy for identifying various lymphoid cells in differentiating cultures after CHIR99021 induction and OP9 coculture induction. R1 representing CD45+ showing CD15, CD56, CD16, or CD94 expression, while none of these markers are present on cells in R2. (TIF 10931 kb) [file 13287_2017_519_MOESM1_ESM.tif]
